# Apicoplast Journey and Its Essentiality as a Compartment for Malaria Parasite Survival

**DOI:** 10.3389/fcimb.2022.881825

**Published:** 2022-04-07

**Authors:** Gagandeep S. Saggu

**Affiliations:** Laboratory of Malaria and Vector Research, National Institute of Allergy and Infectious Diseases (NIH), Rockville, MD, United States

**Keywords:** Malaria, apicoplast, Redox system, IspG enzyme, Fe-S cluster

## Introduction

**Figure 1:**
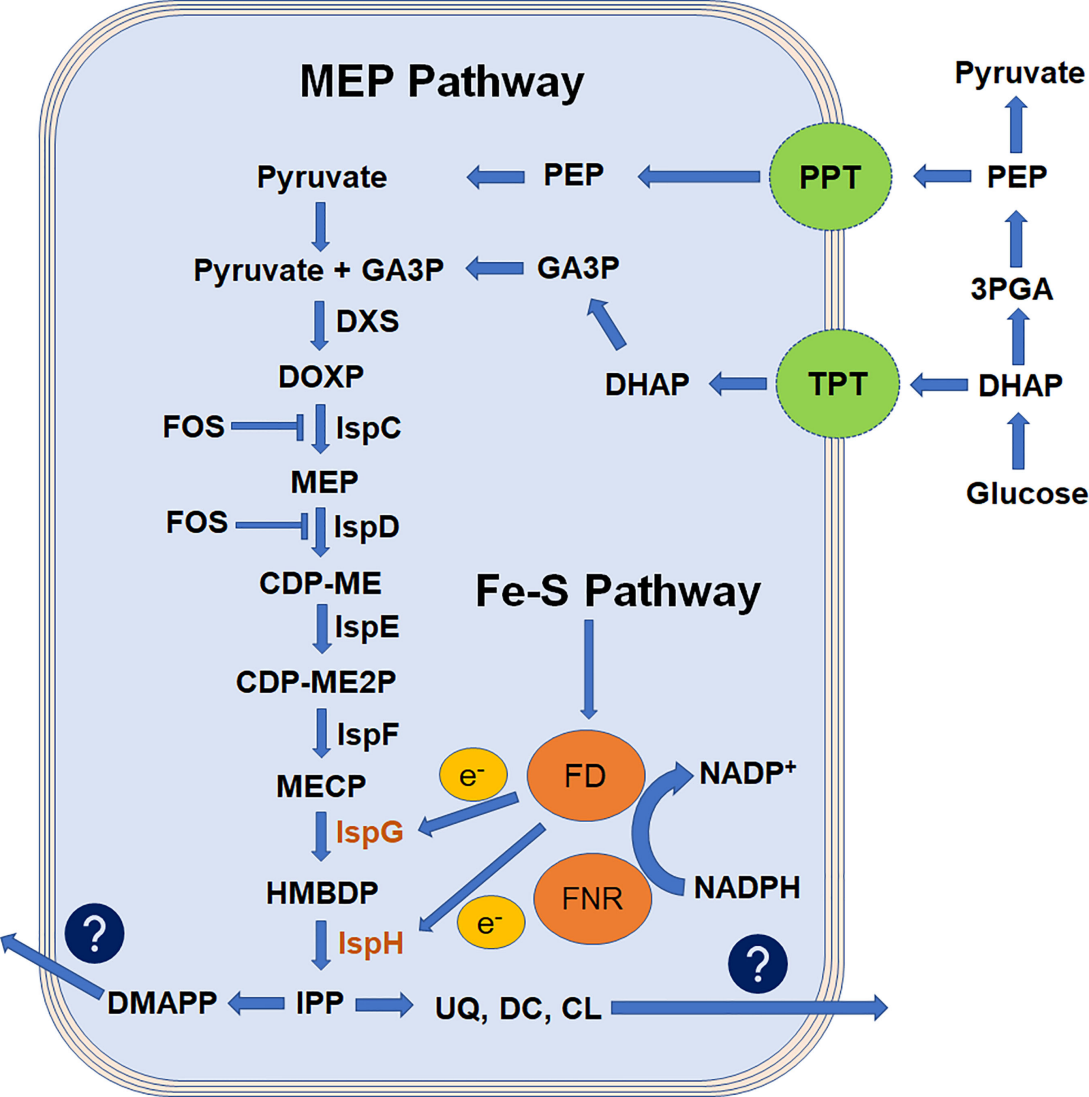
Flowchart representation of apicoplast MEP pathway adapted from Saggu, 2016. Isoprenoids are derived from the basic 5-carbon isoprenoid building blocks of IPP and its isomer, DMAPP. In the MEP pathway, IPP and DMAPP are generated from pyruvate and GA3P and thought to be transported by putative transporter TPT and PPT. Enzymes of this pathway are encoded by parasite nuclear genome and targeted to apicoplast by NEAT sequence. Pathway products are exported out of the apicoplast to parasite cytosol with an unknown mechanism. FOS inhibits the rate-limiting steps of this pathway and blocks the biosynthesis of isoprenoids. Ferredoxin is reduced by the NADPH-dependent enzyme ferredoxin-NADP^+^ reductase and is believed to provide electrons to IspG and IspH enzymes. The MEP pathway is a cascade of enzymatic reactions where inhibition of any step could impede the parasite growth. However, the reduced condition made IspG and IspH enzymes more critical. 3PGA, 3-phospho-glyceraldehyde; CDP-ME, 4-diphosphocytidyl-2C-methyl-d-erythritol; CDP-ME2P, 4-diphosphocytidyl-2C-methyl-d-erythritol-2-phosphate; CL, cholesterol; DC, dolichols; DHAP, dihydroxyacetone phosphate; DMAPP, dimethylallyl pyrophosphate; DXS, 1-deoxy-d-xylose-5-phosphate synthase; DOXP, 1-deoxy-d-xylose-5-phosphate; DXS, 1-deoxy-d-xylulose 5-phosphate synthase; FOS, fosmidomycin; GA3P, glyceraldehyde-3-phosphate; HMBDP, 4-hydroxy-3-methyl-2-(E)-butenyl-4-diphosphate; IPP, isopentenyl-5-pyrophosphate; IspC, 2C-methyl-d-erythritol 4-phosphate synthase; IspD, 4-diphosphocytidyl-2C-methyl-d-erythritol synthase; IspE, 4-diphosphocytidyl-2C-methyl-d-erythritol kinase; IspF, 2C-methyl-d-erythritol 2,4-cyclodiphosphate synthase; IspG, 2C-methyl-d-erythritol 2,4-cyclodiphosphate reductase; IspH, 1-hydroxy-2-methyl-2-(E)-butenyl-4-diphosphate reductase; MECP, 2C-metyl-d-erythritol 2,4-cyclodiphosphate; MEP, 2C-methyl-d-erythritol 4-phosphate; PEP, phosphoenolpyruvate; PPT, phosphoenolpyruvate transporter; TPT, triose-phosphate transporter; UQ, ubiquinones.

The *Plasmodium* parasites belong to the phylum Apicomplexa, and harbor a vital multi membrane organelle known as apicoplast (McFadden et al., 1996; Foth and McFadden, 2003). Various metabolic pathways are functional inside the apicoplast; however, isoprenoid biosynthesis is the essential one of these pathways (Jomaa et al., 1999). The isoprene units are produced *via* two routes in this pathway, mevalonate dependent (MVA; functional in archaea and most eukaryotes) (Katsuki and Bloch, 1967; Lynen, 1967) or mevalonate independent (MEP; functional in bacteria, plant plastid, and apicomplexan parasites) (Arigoni et al., 1997). The MEP pathway is significantly different from the MVA pathway. The malaria parasites completely lost the MVA pathway during the evolutionary process, and the MEP pathway was left as the only option to supplement the isoprenoids requirements (**Figure 1**). Two foundational questions erupted from this process. (1) Why apicoplast-restricted MEP pathway is essential for the malaria parasite and (2) Is the MEP pathway being the only important function of the apicoplast during erythrocytic stages? It is possible that during evolution, the malaria parasite retained the simple energy-efficient MEP route for the biosynthesis of isoprenoids. Whereas, the complex MVA route being extinct made the apicoplast an essential site for this pathway. This opinion became more compelling when MEP pathway inhibition resulted in the immediate death of the parasites, and these parasites were recovered with the external supply of isopentenyl-5-pyrophosphate (IPP) (Yeh and DeRisi, 2011). In addition, gene knockout studies in parasites reveal that other metabolic pathways (type II fatty acid biosynthesis (FASII), haem biosynthesis, and iron–sulfur cluster biosynthesis) are not essential during erythrocytic stages (Seeber, 2003; Ralph et al., 2004). However, the malaria parasite does require the FASII pathway during the liver stage (Vaughan et al., 2009). Genome-based comparison of *Plasmodium* parasites with blood stage-specific parasites *Babesia* and *Theileria* highlights that these parasites lack the gene involved in FASII and haem biosynthesis along with significantly reduced suf genes involved in Fe–S cluster biosynthesis (Brayton et al., 2007; Sato, 2011). This reduction in apicoplast metabolism represents the limited use of apicoplast in these parasites and suggests that the isoprenoid biosynthesis is the prime function of apicoplast during malaria parasites’ erythrocytic (Cassera et al., 2004) and hepatic stages (Sparr et al., 2013). It also implicates that the Fe–S cluster pathway is only required when the MEP pathway is essential for malaria parasites. Additionally, MEP pathway products were also produced and utilized in the early stages of parasite gamete development (Wiley et al., 2015).

What makes apicoplast exclusive for the MEP pathway could be justified with the reducing environment provided inside the apicoplast. It was hypothesized that in the malaria parasite, ferredoxin (Fd) and ferredoxin-NADP+ reductase (FNR) play a central role in the apicoplast maintenance and function (Vollmer et al., 2001; Kehr et al., 2010; Gisselberg et al., 2013). Recent studies described that this system maintains a redox balance in the organelle where it exclusively provides reducing power to various Fe–S cluster-dependent proteins functional inside the apicoplast without having any role in apicoplast maintenance (Swift et al., 2022). Further interrogation of MEP pathways enzymes states their specific requirement of reducing environment, divalent cations, and electron transfer through NADP that could only be possible inside the apicoplast. Another point of evidence provided by the failed attempt to create a complete MEP pathway in *Saccharomyces cerevisiae*, later attributed to the lack of a suitable redox environment (Partow et al., 2012). Studies described so far specify that the sole function of the apicoplast is to provide a reduced compartment for successful completion of the MEP pathway, suggesting that the site of pathway execution is more fundamental.

The structures of the MEP pathway enzymes were predicted from various microorganisms, plants, and malaria parasites (Umeda et al., 2011; Handa et al., 2013; Rekittke et al., 2013; O'Rourke et al., 2014; Umeda et al., 2015), providing an insight into the enzyme kinetics (Saggu et al., 2016). It highlights that the divalent cation and electron transfer process are crucial for the substrate binding and for the enzyme conformational changes. For example, the enzymatic activity of IspG (4-hydroxy-3-methylbut-2-en-1-yl diphosphate synthase, also named GcpE) and IspH (4-hydroxy-3-methyl-2-(E)-butenyl-4-diphosphate reductase (lytB)) enzymes are solely dependent on the reducing environment (**Figure 1**). The IspG enzyme involved in the penultimate step of the MEP pathway catalyzes the conversion of cyclic 2-C-methyl-d-erythritol-2,4-cyclodiphosphate (MECP) molecule to aliphatic 4-hydroxy-3-methyl-2-(E)-butenyl-4-di phosphate (HMBDP) in a stepwise process. Here, IspG enzyme core provides a binding site for MECP substrate on a TIM barrel domain followed by the [4Fe–4S] cluster binding to the conserved cysteine residues aligned in the cap region (Saggu et al., 2017). This binding is highly dependent on a redox reaction where electrons required for the conversion process are thought to be supplied by ferredoxin through [4Fe–4S] clusters (Röhrich et al., 2005; Pala et al., 2019). This close confirmation lets [4Fe–4S] cluster come in proximity to the MECP substrate, and the electron transfer process converts the cyclic MECP to an aliphatic HMBDP product. This HMBDP is then converted to the IPP and DMAPP by IspH enzyme in a three-step conversion process: (i) removal of a hydroxyl group, (ii) transfer of two electrons from the [4Fe–4S] cluster, and (iii) the protonation of an intermediate allylic anion (Laupitz et al., 2004). IspH enzyme has two domains, a LytB domain for the binding of HMBDP and a [4Fe–4S] cluster-binding domain-like IspG. In this process, the Fe–S cluster biosynthesis pathway has an essential function to provide [4Fe–4S] clusters for the IspG and IspH enzyme functionality (**Figure 1**) (Pala et al., 2018; Swift et al., 2022). In our study, the IspG enzyme expressed and purified in laboratory conditions was not functional until a [4Fe–4S] cluster to this oxidized IspG was not transferred in an artificially reduced environment (Pala et al., 2016; Saggu et al., 2017; Pala et al., 2019).

An interesting *Plasmodium* parasite line was engineered where isoprenoid precursors are produced using the MVA route with an external supply of mevalonate (Swift et al., 2020). However, IspG and IspH enzyme deletion stunted these parasites’ growth in the absence of mevalonate (Swift et al., 2022). Indirect involvement of IspG enzyme was also studied where enzyme inhibition led to the accumulation of MECP substrate resulting in retrograde signaling, altering the nuclear architecture and functional dynamics (Xiao et al., 2012). Studies described here support the view that during evolution, parasites were unable to replace the functionality and environmental condition facilitated by the apicoplast and that seems to be the fundamental reason to let apicoplast persist inside the parasite. In the future, it is possible to witness a parasite lacking this compartment; however, that will either require a parasite growing without isoprene units or a more efficient organelle facilitating equally good environmental conditions.

## Apicoplast and MEP Pathway Enzymes as Drug Targets

The apicoplast division is tightly regulated with a dynamin-like protein equivalent to plant plastids (Striepen et al., 2000; van Dooren et al., 2009) and might have a physical connection between their genome and centrioles in the cytoplasm, which neatly ensures the crucial portioning of the organelle genome into daughter cells (Francia et al., 2012). A close association between apicoplast and mitochondria was reported in malaria parasites, emphasizing its correlation with other organelles during its progression to the next parasitic stage (van Dooren et al., 2005). During metabolic processes, various steps of the haem biosynthesis pathway jump between apicoplast and mitochondria giving a hint about the transport between these organelles. However, this phenomenon is not validated with the presence of any transporter.

The apicoplast genome consists of a reduced 35-kb circular molecule that encodes for the limited housekeeping functions (approximately 30 proteins, tRNAs, and some RNAs) (Fichera and Roos, 1997; Saxena et al., 2012). In contrast, more than 500 proteins are encoded and translocated from the parasite cytosol to the apicoplast with the help of a bipartite leader sequence (Ralph et al., 2004). These proteins are known as nuclear-encoded apicoplast-targeted (NEAT) proteins and are involved in various metabolic pathways (Parsons et al., 2007). The apicoplast housekeeping processes are significantly different from the host, suggesting their value as a target for the development of potent and safe therapies for malaria treatment. Inhibition of these processes with antibiotics limits the division of apicoplast and its progression into subsequent stages, ultimately killing the apicoplast-free parasite after 48 h, known as the delayed death effect (Kennedy et al., 2019). However, these apicoplast-free parasites could be revived with an external supply of IPP, the final product of the MEP pathway (Yeh and DeRisi, 2011). This delayed death phenomenon also limits the use of these inhibitors in the case of severe malaria.

Metabolic pathway enzymes were also explored as putative drug targets, and initial studies reported FASII enzymes as a promising target (Waller et al., 2003). However, later studies questioned the essentiality of this pathway during erythrocytic stages (Vaughan et al., 2009). Whereas, inhibition of MEP pathway enzymes causes the immediate death of parasites. The IspC (DXP reductoisomerase/DXR) and IspD [2-C-methyl-d-Erythritol 4-phosphate cytidylyltransferase (YgbP)] enzymes are studied extensively as a target of natural antibiotic fosmidomycin (Fos) (Jomaa et al., 1999; Zhang et al., 2011; Imlay et al., 2015; Saggu et al., 2018), and these studies also made it to the clinical stages (Wiesner and Jomaa, 2007). However, drawbacks like low absorption and shorter half-life (Kuemmerle et al., 1985) did not allow it to stand alone for further validation, and hence, it was used in combination with other antimalarials (Na-Bangchang et al., 2007). To overcome this limitation, different research groups synthesized various analogs of Fos with chemical modification without any success or activity equal to Fos *in vivo*. During the inhibition process, Fos competes for the binding on the active site of the IspC enzyme, and *P. falciparum* had one strain develop resistance against it by higher production of IspC substrate, 1-deoxy-d-xylose-5-phosphate (DXP) (Wang et al., 2018). Based on the similarity with prokaryotic and plant MEP pathways, other enzymes of the MEP pathway were also mapped and studied as a target for various inhibitors (Wang et al., 2010; Saggu et al., 2016). This pathway provides multiple opportunities to develop a valuable combination therapy that could be either the various enzymes of this pathway or the redox environment created for the functioning of this pathway (Saggu et al., 2016; Kimata-Ariga and Morihisa, 2022).

## Conclusion

Apicoplast origin with secondary endosymbiosis resulted as a continuous degenerative process for this relict essential plastid. This process started with losing its autonomous power by losing its photosynthetic ability followed by genome translocation to the host cell (McFadden and van Dooren, 2004), making it exclusively host-cell dependent. For establishing a symbiotic relationship, the host cell reduced its metabolic functionality, which was equivalent to the apicoplast, and with progression, these metabolic pathways became essential for the host. However, continuous degenerative processes made these pathways more concise and restricted to a few enzymatic reactions. In the present form, the only essential function performed by the apicoplast is the MEP pathway. It requires the Fe–S cluster pathway to synthesize and transfer the [4Fe–4S] cluster to various enzymes in a redox environment created by Fd and FNR (Swift et al., 2022). As discussed earlier, these enzymes are NEAT proteins but their import mechanism is not well understood. In this scenario, it could be hypothesized that inhibition of the import of these proteins into the apicoplast could also lead to outcomes like MEP pathway inhibition. In the future, with this continuous degenerative process apicoplast replacement may be possible with another compartment providing environmental conditions suitable for the MEP pathway enzyme functionality.

## Author Contributions

The author confirms being the sole contributor of this work and has approved it for publication.

## Funding

This study was supported by the Intramural Research Program of the National Institutes of Health, National Institute of Allergy and Infectious Diseases. The funders had no role in study design, data collection, analysis, decision to publish, or preparation of the manuscript.

## Conflict of Interest

The author declares that the research was conducted in the absence of any commercial or financial relationships that could be construed as a potential conflict of interest.

## Publisher’s Note

All claims expressed in this article are solely those of the authors and do not necessarily represent those of their affiliated organizations, or those of the publisher, the editors and the reviewers. Any product that may be evaluated in this article, or claim that may be made by its manufacturer, is not guaranteed or endorsed by the publisher.
